# Assessment of correlation between hand-wrist maturation and cervical vertebral maturation: a fractal analysis study

**DOI:** 10.1186/s12903-023-03483-0

**Published:** 2023-10-26

**Authors:** İlknur Eninanç, Zeynep Çoban Büyükbayraktar

**Affiliations:** 1 Department of Oral and Maxillofacial Radiology, Faculty of Dentistry, Sivas Cumhuriyet University, Sivas, Turkey; 2 Department of Orthodontics, Faculty of Dentistry, Sivas Cumhuriyet University, Sivas, Turkey

**Keywords:** Hand-wrist maturation, Cervical vertebral maturation, Fractal dimension, Trabecular bone

## Abstract

**Background:**

To investigate whether fractal dimension (FD) measurements from hand-wrist radiographs and lateral cephalometric radiographs are correlated with each other and with skeletal maturation stages.

**Methods:**

In this retrospective study conducted on hand-wrist and lateral cephalometric radiographs obtained from patients between 2017 and 2023, hand-wrist maturation stages (HWMS) and cervical vertebral maturation stages (CVMS) of 144 subjects (6 to 17 years of age) were assessed radiographically. The participants were divided into nine groups (n = 16 each) based on HWMS. Fractal analysis was performed on the radiographs of the radius, the middle finger phalanges (proximal, medial and distal), and the cervical vertebral bodies (C2, C3, C4). Mean and standard deviation values, Spearman’s and Pearson correlation analyses, one-way ANOVA, Kruskal-Wallis H tests and Mann-Whitney-U test were used to evaluate the data.

**Results:**

Positive correlations were found between the FD values of the radius and HWMS or CVMS *(r = .559, P = .001, r = .528 P = .001* respectively*).* The FD values of the radius were positively correlated with those of all cervical vertebrae (C2, C3, C4), proximal and medial phalanges as well as age. FD values measured from the proximal phalanx, medial phalanx and radius showed significant differences among both HWMS and CVMS *(P < .05).* HWMS was strongly correlated with CVMS *(r = .929, P = .001*). Age was strongly correlated with HWMS (*r = .795, P = .001*) and CVMS *(r = .756, P = .001)*. There was a significant difference in terms of age distribution among HWMS and CVMS *(P < .05)*.

**Conclusions:**

FD measurements on hand-wrist radiographs can provide useful information for the assessment of skeletal maturation stage. Especially, FD measurements from the radius are important and more reliable to predict skeletal maturation stage.

## Background

Growth and development follow a natural course and involve periods of accelerated growth known as growth spurts [1]. Chronological age, dental and skeletal development, sexual maturation and increases in height and body weight are used to determine growth stages [2, 3]. In dentistry, it may sometimes be necessary to predict growth stage for diagnostic and prognostic purposes especially in surgical, pedodontic and orthodontic practices [4]. In orthodontics, the growth and development of the jaw, growth spurts, and physiological facial growth are evaluated for the treatment of skeletal anomalies. This evaluation is undertaken with the aim to provide the most appropriate treatment according to the individual’s developmental stages and to achieve maximum success in the shortest time possible. Additionally, knowledge of the growth rate and remaining growth potential is important in preventing relapses that may occur after the treatment [5–7]. A variety of radiographs are used to determine growth and development of the growing patient for orthodontic purposes. The hand-wrist maturation (HWM) analysis is a traditional and widely used method for the assessment of skeletal maturation [8]. However, the use of the HWM method is limited by a number of concerns such as sexual or ethnic dimorphism and the requirement for additional exposure of patients to radiation [9, 10]. Consequently, the cervical vertebral maturation (CVM) staging on lateral cephalometric radiographs has been introduced as an alternative to the HWM method [11, 12]. Previous studies have shown that the hand-wrist maturation stages (HWMS) and cervical vertebral maturation stages (CVMS) are strongly correlated [13–16]. Also, it has been reported that the CVM method reliably predicts pubertal growth, and is highly consistent with the HWM method in predicting skeletal maturation in adolescence [17, 18].

Fractal analysis is a method used to determine the complexity of the shapes or structural patterns within the bone, which is numerically expressed as fractal dimension (FD) [19]. Information about the 3D structure of the bone can be obtained through two-dimensional images, and fractal analysis enables detection of details that may be missed on radiographs [4, 13, 20]. Changes in the trabecular bone pattern have been demonstrated in several studies using fractal analysis [21–23].

As the complexity of the bone pattern increases, fractal dimension increases, and while a greater FD value represents denser bone structure with fewer pores within the bone, a lower FD value indicates larger pores and greater loss of bone [24]. The fractal analysis method was used in this study to measure the extent of bone mineralization due to its cost-effectiveness and no requirement of additional irradiation.

In this study, changes in trabecular structure were investigated through fractal dimension measurements from various regions on hand-wrist radiographs and lateral cephalometric radiographs in individuals at different stages of growth and development. The aims of this study were to morphologically examine the correlation of both HWMS and CVMS with FD values measured on the hand-wrist radiographs and lateral cephalometric radiographs; the correlation of FDs among the measured sites on both radiographs; and significance of differences in FDs among the 9 HWMS and among the 6 CVMS for each of the measured sites. To the best of the authors’ knowledge, this is the first study to compare FD measurements from both hand-wrist and lateral cephalometric radiographs.

## Methods

This study had a retrospective design. Ethics approval for the study was obtained from the Institutional Review Board of Sivas Cumhuriyet University (ID: 2022-11/13). The study was conducted in accordance with the principles laid out in the Declaration of Helsinki. Written or verbal informed consent were obtained from the patients and their legal guards separately. Hand-wrist and lateral cephalometric radiographs obtained from patients aged 6 to 17 years between 2017 and 2023 at the Department of Orthodontics, Sivas Cumhuriyet University were used for this study, and no additional radiographs were acquired.

Patients with current or past orthodontic treatment, any metabolic bone disease, history of treatment that could affect bone metabolism, previous trauma to the face, congenital or acquired malformations in the regions examined in this study as well as radiographs with artifacts or positioning errors, and radiographs that did not allow for FD measurement were excluded from the study. Among the hand-wrist and lateral cephalometric radiographs, those acquired at a fixed dose and exposure settings of 85 kVp and 13 mA were included in the study. Of the 374 hand-wrist and lateral cephalometric radiographs from 187 patients, 288 radiographs from 144 patients meeting the eligibility criteria were included in the study.

It was estimated that a sample size of 144 subjects (72 girls, 72 boys) would be needed for the study, assuming f = 0.551, α = 0.05, β = 0.10, and power (1-β) = 0.95 [20]. Based on this sample size, the power of test calculated with G*Power 3.1 was 0.95.

### Radiographic evaluation

Hand-wrist and lateral cephalometric radiographs of the patients were acquired on the same day using a digital radiography device (Instrumentarium OP200 D, Instrumentarium Dental, Finland). As the majority of the general population is right-handed, and the dominant hand is more likely to be injured than the non-dominant (left) hand, left hand and wrist radiographs were used for determining maturation stage [25]. Hand-wrist radiographs were obtained using a standard protocol, with the long axis of the third finger of the left hand aligned with the forearm.

HWMS staging on hand-wrist radiographs was performed by an orthodontist (Z. ÇB) with 9 years of clinical experience using the classification described Björk [26] and Grave-Brown [27]. The subjects were divided into 9 HWM stages:

- *HWMS-1 (PP2)*: the epiphyseal and diaphyseal widths of the proximal phalanx of the index finger (PP2) are equal.
- *HWMS-2 (MP3)*: the epiphyseal and diaphyseal widths of the middle phalanx of the middle finger (MP3) are equal.
- *HWMS-3 (Pisi-H1-R)*: Pisi; ossification of the pisiform bone, H1; ossification of the hamular process of the hamate bone, R; Radius, the epiphysis of the radius is equal in width to the diaphysis.
- *HWMS-4 (S-H2)*: S; initial mineralization of ulnar sesamoid bone of the metacarpophalangeal joint of the hamatum is evident, H2; ossification of the hamular process of the hamate bone is prominent.
- *HWMS-5 (MP3cap-PP1cap-Rcap)*: MP3cap; cap-shaped epiphysis covers diaphysis at the middle phalanx of the third finger, PP1cap; capping of the diaphysis by epiphysis in the proximal phalanx of the thumb, Rcap; capping of the diaphysis by epiphysis in the radius.
- *HWMS-6 (DP3u)*: fusion of the diaphysis and epiphysis of the distal phalanx of the middle finger (DP3).
- *HWMS-7 (PP3u)*: fusion of the diaphysis and epiphysis of the proximal phalanx of the middle finger (PP3).
- *HWMS-8 (MP3u)*: union of the diaphysis and epiphysis in the middle phalanx of the third finger (MP3).
- *HWMS-9 (Ru)*: fusion of the diaphysis and epiphysis of the radius (R); complete ossification is observed.

CVM staging was performed by the same orthodontist on the lateral cephalometric radiographs using Hassel and Farman [11] classification. The subjects were divided into 6 CVMS:

- *Cervical Vertebral Maturation Stage 1 (CVMS1)*: Initiation stage. Adolescent growth is just beginning and 80–100% of adolescent growth is expected. The cervical 2 (C2), cervical 3 (C3) and cervical 4 (C4) vertebral bodies are triangular and the upper margins are tapered posteriorly.
- *Cervical Vertebral Maturation Stage 2 (CVMS2)*: Stage of acceleration, where adolescent growth accelerates. 65–85% growth is expected. Concavity emerges at the inferior borders of C2 and C3. The inferior border of C4 is flat. The C3 and C4 vertebral bodies are slightly rectangular.
- *Cervical Vertebral Maturation Stage 3 (CVMS3)*: Stage of transition. Growth is fastest. 25 to 65% growth is expected. The inferior marginss of C2 and C3 have a well-defined concavity. Concavity develops at the inferior margin of C4. C3 and C4 are rectangular.
- *Cervical Vertebral Maturation Stage 4 (CVMS4)*: Stage of deceleration. Growth is much slower. 10 to 25% growth is expected. Distinct concavities are present at the inferior margins of C2, C3 and C4. The shape of C3 and C4 is almost square.
- *Cervical Vertebral Maturation Stage 5 (CVMS5)*: Maturation stage. Growth is negligible. Expected growth rate is 5 to 10%. The inferior margins of C2, C3 and C4 are distinctly concave. C3 and C4 are square-shaped.
- *Cervical Vertebral Maturation Stage 6 (CVMS6)*: Stage of completion. Growth is complete. No further growth is expected. The inferior margins of C2, C3 and C4 show deep concavities. C3 and C4 have a square shape or horizontal dimension is smaller than vertical dimension.

FD measurements on hand-wrist and lateral cephalometric radiographs were performed by a dentomaxillofacial radiologist (İ.E.) with a total of 12 years of clinical experience including 6 years of specialist experience in maxillofacial radiology who was blinded to the chronological age and sex of the patients and other variables tested.

Repeat fractal analyses were conducted by the same dentomaxillofacial radiologist twice at 2-week intervals on randomly selected hand-wrist (n = 36, 25%) and cephalometric radiographs (n = 36, 25%).

### Display features

For FD analysis, all digital radiographs were reviewed by the dentomaxillofacial radiologist on a 64-bit LCD screen (Lenovo IdeaPad Z500 Intel Core i5) with 15.6-inch LED (Light Emitted Diode) backlight and 1366 × 768-pixel resolution in a semi-lit, quiet room.

### Image Processing (Fractal Dimension Analysis)

Hand-wrist radiographs (size: 2212 × 2304 pixels) and lateral cephalometric radiographs (size: 2836 × 2304 pixels) were used in this study.

The Windows version of ImageJ 1.53k image analysis software bundled with 64-bit Java downloaded from the National Institute of Health Image website (https://imagej.nih.gov) was used for FD analysis. The images were saved in high-resolution JPEG format. The box counting method described by White and Rudolph [28] was used for FD analysis.

### ROI (region of interest) selection

All radiographs were reviewed to select the appropriate ROI size. In total, four ROIs were selected for FD analysis on hand-wrist radiographs of the left hand and wrist excluding cortical margins:

1- Epiphyseal/diaphyseal line of the proximal phalanx of the third finger (80 × 40 pixels),

2- Epiphyseal/diaphyseal line of the medial phalanx of the third finger (80 × 40 pixels),

3- Epiphyseal/diaphyseal line of the distal phalanx of the third finger (50 × 30 pixels),

4- Epiphyseal/diaphyseal line of the radius (200 × 50 pixels).

Three ROIs (60 × 60 pixels) were selected for FD analysis on lateral cephalometric radiographs excluding cortical margins:

1- C2 vertebral body,

2- C3 vertebral body, and.

**Fig. 1:**
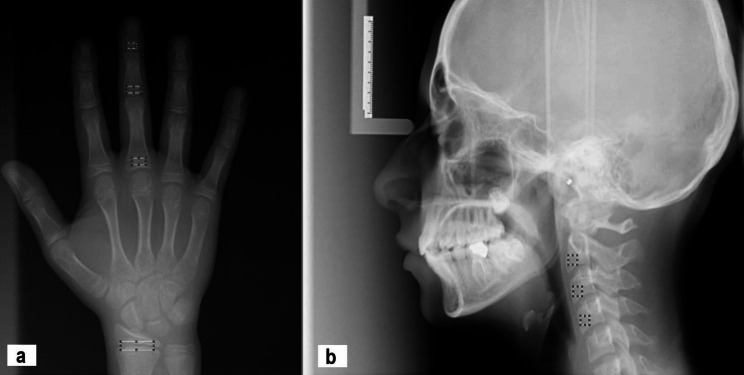
(**a**) Regions of interest (ROIs) from 4 different sites on hand-wrist radiographs (proximal, distal, medial phalanx and radius), (**b**) ROIs from 3 different sites on the cephalometric image (C2, C3 and C4 cervical vertebral bodies)

3- C4 vertebral body (Fig. 1).

**Fig. 2:**
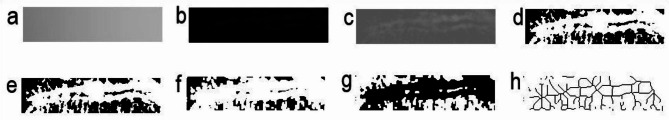
(**a**) Blurred image, (**b**) Subtraction of blurred image from the original image, (**c**) Addition of 128 grayscale value, (**d**) Conversion of image colors, (**e**) Erosion, (**f**) Dilation, (**g**) Inversion, (**h**) Skeletonization

The ROIs selected for FD analysis were cropped and duplicated (Fig. 2). To preserve large density variations on the image, Gaussian filter (sigma = 35 pixels) was applied to blur extremely or moderately bright areas in the image resulting from uneven thickness of the soft tissue covering the bone (Fig. 2/a). Next, the blurred image was subtracted from the original image (Fig. 2/b) and 128 grayscale value was added (Fig. 2/c). Areas with variable brightness enabled differentiation between bone marrow and trabecular structure. Then, the image was converted to a black and white image using the “Threshold” tool, rendering the outlines of the trabecular bone and bone marrow distinguishable (Fig. 2/d). The noise of the image was eliminated using the “Erode” tool (Fig. 2/e), and then, the “Dilate” tool was used to add pixels to the boundaries of the objects in the image, making them more visible (Fig. 2/f). Image colors were inverted (black to white and vice versa) to reveal the outline of the trabecular bone using the “Invert” tool (Fig. 2/g). The foreground areas on the binary image were reduced to a skeletal remnant using the “Skeletonize” tool (Fig. 2/h). Fractal dimensions were calculated for the outlines of the trabeculae with the “Analyze” tool. Then, the image was divided into squares of 2, 3, 4, 6, 8, 12, 16, 32 and 64 pixels. The number of squares with trabeculae and the total number of squares in the image were calculated for all pixels of different size. The values obtained were plotted on a logarithmic scale. The slope of the line of best fit provided the FD value indicating the complexity degree of the structure.

### Statistical method

SPSS Statistics, version 23.0 (IBM Corp., Armonk, NY) was used for statistical analyses of the study data. The ordinal categorical variables (HWMS, CVMS) were evaluated using non-parametric tests. For other quantitative data, normality of distribution was checked using Shapiro-Wilk test, and analyses were performed [29, 30]. Correlations of FD measurements from the ROIs with HWMS or CVMS were examined using Spearman’s correlation analysis. Correlations between FD values measured from hand-wrist and lateral cephalometric radiographs were investigated using Pearson correlation analysis. Correlations of chronological age with HWMS, CVMS and FD values from ROIs were examined using Spearman’s correlation analysis (Spearman’s rank-order correlation coefficient). The correlation coefficient defined as very strong (0.90-1), strong (0.70–0.89), moderate (0.50–0.69), weak (0.30–0.49) and very weak (0.00-0.29) [29].

One-way analysis of variance (ANOVA) was used to compare FD values from the ROIs by HWMS and CVMS. Kruskal-Wallis H test and Dunn’s multiple comparison test were used to compare age distribution among HWMS and CVMS. Post hoc tests were performed, including Tukey’s test, for the data that met homogeneity of variance assumption and Tamhane’s T2 test for the data that did not. Mann-Whitney-U test was used to analyze age-related differences between sexes. Intra-correlation coefficients were calculated to determine intra-observer agreement. The statistically significant level was set at 0.05.

## Results

A total of 144 subjects (72 boys, 72 girls) were included in the study. The sex distribution of all groups was equal among the 9 HWMS groups.

Hand-wrist radiographs of 144 patients divided into 9 HWM stages were included in this study, and fractal analysis was performed for 576 ROIs (144 × 4) on these radiographs. In addition, lateral cephalometric radiographs of the same patients categorized into 6 CVMS were studied, and FD measurements were obtained from 432 (144 × 3) ROIs.

HWMS showed moderate positive correlations with FD values of the radius and weak positive correlations with FD of the proximal phalanx, medial phalanx, C2 and C3, which were all significant (Table 1).

CVMS showed a moderate positive correlation with the FD of the radius and weak positive correlations with the FD of the proximal phalanx, medial phalanx and C3 (all significant) (Table 1).

**Table 1:** Correlations among HWMS, CVMS, fractal dimension measurements and age

		HWMS	CVMS	Proximal Phalanx	Medial Phalanx	Distal Phalanx	Radius	C2	C3	C4	Age	
HWMS	r	1.000^+^	0.929^+**^	0.200^+*^	0.323^+**^	0.147^+^	0.559^+**^	0.187^+*^	0.236^**^	0.110^+^	0.795^+**^	
	P	.	0.001	0.016	0.001	0.079	0.001	0.025	0.004	0.188	0.001	
CVMS	r		1.000^+^	0.172^+*^	0.278^+**^	0.151^+^	0.528^+**^	0.150^+^	0.224^+**^	0.124^+^	0.756^+**^	
	P		.	0.039	0.001	0.071	0.001	0.073	0.007	0.140	0.001	
Proximal Phalanx	r			1.000	0.414^**^	0.210^*^	0.318^**^	− 0.061	0.021	− 0.011	− 0.035^+^	
	P			.	0.001	0.012	0.001	0.464	0.798	0.895	0.679	
Medial Phalanx	r				1.000	0.322^**^	0.328^**^	0.002	0.173^*^	0.057	0.114^+^	
	P				.	0.001	0.001	0.982	0.038	0.494	0.173	
Distal Phalanx	r					1.000	0.075	− 0.081	− 0.029	− 0.100	0.116^+^	
	P					.	0.370	0.336	0.734	0.232	0.165	
Radius	r						1.000	0.182^*^	0.370^**^	0.377^**^	0.403^+**^	
	P						.	0.029	0.001	0.001	0.001	
C2	r							1.000	0.454^**^	0.367^**^	0.245^+**^	
	P							.	0.001	0.001	0.003	
C3	r								1.000	0.635^**^	0.253^+**^	
	P								.	0.001	0.002	
C4	r									1.000	0.123^+^	
	P									.	0.142	
Age	r										1.000	
	P											

^+^Spearman’s rank- order correlation coefficient

Pearson’s correlation coefficient

**Correlation is significant at the 0.01 level (2-tailed)

*Correlation is significant at the 0.05 level (2-tailed)

Table 1 shows correlations between FD measurements obtained from hand-wrist radiographs and lateral cephalometric radiographs. In summary:

- FD of the radius was positively weakly correlated with FD of the proximal, medial phalanx, C2, C3 and C4 (*all significant)*.
- Proximal phalanx FD was weakly correlated with FD of the medial phalanx, or distal phalanx (*all significant)*.
- Medial phalanx FD was weakly correlated with FD of the distal phalanx and C3 *(all significant)*.
- C2 FD showed weakly positive correlations with C3 and C4 FD values *(both significant)*.
- C3 FD showed a moderate positive correlation with C4 FD.

HWMS was very strongly positive correlated with CVMS (Table 1).

Age was strongly correlated with HWMS (*r = .905, P = .001 for girls; r = .824, P = .001 for boys*) and CVMS *(r = .861, P = .001 for girls; r = .783, P = .001 for boys)*. Age was weakly correlated with FD measurements of the radius, C2 and C3 (Table 1).

FD values from the proximal phalanx, medial phalanx and radius were significantly different among HWMS and CVMS. There was a significant difference in terms of age distribution among HWMS and CVMS. (Tables 2 and 3).

**Table 2:** Comparison of fractal dimension measurements and age among HWMS

	N	Proximal phalanx	Medial phalanx	Distal phalanx	Radius	C2	C3	C4	Age	
		Mean ± SD	Mean ± SD	Mean ± SD	Mean ± SD	Mean ± SD	Mean ± SD	Mean ± SD	Mean ± SD	
**HWMS-1**	16	1.270 ± 0.032	1.264 ± 0.042	1.312 ± 0.045	1.337 ± 0.043	1.535 ± 0.041	1.574 ± 0.064	1.559 ± 0.038	8.375 ± 2.06	
**HWMS-2**	16	1.266 ± 0.043	1.253 ± 0.040	1.333 ± 0.054	1.322 ± 0.050	1.530 ± 0.054	1.539 ± 0.062	1.531 ± 0.068	11.000 ± 2.00	
**HWMS-3**	16	1.249 ± 0.061	1.247 ± 0.039	1.313 ± 0.042	1.325 ± 0.055	1.544 ± 0.077	1.553 ± 0.076	1.531 ± 0.072	11.563 ± 2.06	
**HWMS-4**	16	1.228 ± 0.045	1.241 ± 0.056	1.313 ± 0.032	1.334 ± 0.059	1.542 ± 0.060	1.557 ± 0.046	1.549 ± 0.064	12.125 ± 1.08	
**HWMS-5**	16	1.254 ± 0.055	1.242 ± 0.033	1.316 ± 0.056	1.344 ± 0.056	1.559 ± 0.050	1.580 ± 0.055	1.543 ± 0.055	12.625 ± 1.54	
**HWMS-6**	16	1.266 ± 0.047	1.257 ± 0.050	1.303 ± 0.039	1.384 ± 0.033	1.566 ± 0.051	1.580 ± 0.047	1.554 ± 0.060	13.313 ± 1.30	
**HWMS-7**	16	1.288 ± 0.046	1.277 ± 0.062	1.316 ± 0.073	1.382 ± 0.044	1.562 ± 0.094	1.581 ± 0.061	1.551 ± 0.063	14.188 ± 1.22	
**HWMS-8**	16	1.279 ± 0.053	1.307 ± 0.057	1.348 ± 0.043	1.383 ± 0.042	1.548 ± 0.069	1.595 ± 0.060	1.540 ± 0.070	15.063 ± 1.34	
**HWMS-9**	16	1.295 ± 0.043	1.320 ± 0.067	1.353 ± 0.075	1.442 ± 0.037	1.569 ± 0.062	1.597 ± 0.061	1.587 ± 0.046	15.875 ± 1.40	
P		0.005* ^*c*^	0.001* ^*ab*^	*0.101*	0.001* ^*defg*^	*0.644*	*0.109*	*0.280*	*0.001* ^*+*^ * ^*hijkl*^	

**One-way ANOVA**, ^**+**^**Kruskal-Wallis H Test**,

**Post-hoc tests; Tamhane’s T2 test**: ^a^HWMS-9 vs. HWMS-3, HWMS-4, and HWMS-5 (P = .035, P = .044, P = .016 respectively); ^b^HWMS-5 vs. HWMS-8 (P = .025)

**Tukey**: ^c^HWMS-4 vs. HWMS-7 and HWMS-9 (P = .016, P = .004 resoectively); ^d^HWMS-1 vs. HWMS-9 (P = .000), ^e^HWMS-2 vs. HWMS-6, HWMS-7, HWMS-8 and HWMS-9 (P = .011, P = .016, P = .014, and P = .001, respectively); ^f^HWMS-3 vs. HWMS-6, HWMS-7, HWMS-8 and HWMS-9 (P = .019, P = .028, P = .024, and P = .001, respectively) ; ^g^HWMS-9 vs. HWMS-4, HWMS-5, HWMS-6, HWMS-7, and HWMS-8 (P = .001, P = .001, P = .025, P = .017, P = .020 respectively)

Dunn’s test:^h^HWMS-1 vs. HWMS-5, HWMS-6, HWMS-7, HWMS-8, and HWMS-9 (P = .016, P = .001, P = .001, P = .001, P = .001, respectively), ^i^HWMS-2 vs. HWMS-7, HWMS-8, and HWMS-9 (P = .005, P = .001, P = .001, respectively), ^j^HWMS-3 vs. HWMS-7, HWMS-8, and HWMS-9 (P = .045, P = .001, P = .001, respectively), ^k^HWMS-4 vs. HWMS-8, and HWMS-9 (P = .005, P = .001, respectively), ^l^HWMS-5 vs. HWMS-9 (P = .004).

*Significant at P < .05

*HWMS: Hand-Wrist Maturation Stages*

**Table 3:** Comparison of fractal dimension measurements and age among CVMS.

	N	Proximal phalanx	Medial phalanx	Distal phalanx	Radius	C2	C3	C4	Age
		Mean ± SD	Mean ± SD	Mean ± SD	Mean ± SD	Mean ± SD	Mean ± SD	Mean ± SD	Mean ± SD
**CVMS-1**	5	1.272 ± 0.028	1.259 ± 0.043	1.300 ± 0.069	1.341 ± 0.038	1.542 ± 0.027	1.580 ± 0.046	1.564 ± 0.029	8.800 ± 2.38
**CVMS-2**	19	1.253 ± 0.047	1.249 ± 0.049	1.321 ± 0.041	1.324 ± 0.046	1.548 ± 0.057	1.549 ± 0.074	1.543 ± 0.057	9.579 ± 2.67
**CVMS-3**	35	1.255 ± 0.054	1.258 ± 0.042	1.313 ± 0.043	1.332 ± 0.058	1.533 ± 0.060	1.558 ± 0.057	1.538 ± 0.068	11.571 ± 1.70
**CVMS-4**	28	1.252 ± 0.051	1.246 ± 0.041	1.323 ± 0.048	1.352 ± 0.053	1.562 ± 0.057	1.571 ± 0.058	1.536 ± 0.053	12.536 ± 1.47
**CVMS-5**	40	1.290 ± 0.046	1.282 ± 0.067	1.331 ± 0.063	1.387 ± 0.044	1.551 ± 0.077	1.590 ± 0.061	1.565 ± 0.061	14.450 ± 1.53
**CVMS-6**	17	1.268 ± 0.047	1.312 ± 0.062	1.337 ± 0.069	1.424 ± 0.050	1.571 ± 0.058	1.589 ± 0.058	1.562 ± 0.065	15.647 ± 1.41
**P**		0.015*^a^	0.001*^b^	0.544	0.001*^cd^	0.351	0.096	0.279	.001*^+efgh^

**One-way ANOVA**, ^**+**^**Kruskal-Wallis H Test**,

**Post-hoc tests; Tukey**^a^CVMS-5 vs. CVMS-3, and CVMS-4 (P = .034, P = .026 respectively); ^b^CVMS-6 vs. CVMS-2, CVMS-3, and CVMS-4 (P = .009, P = .013, P = .002 respectively); ^c^CVMS-6 vs. CVMS-1, CVMS-2, CVMS-3, and CVMS-4 (P = .019, P = .001, P = .001, P = .001 respectively); ^d^CVMS-5 vs. CVMS-2, and CVMS-3 (P = .001, P = .001)

**Dunn’s test**:^e^CVMS-1 vs. CVMS-5 and CVMS-6 (P = .001, P = .001, respectively), ^f^CVMS-2 vs. CVMS-4, CVMS-5 and CVMS-6 (P = .049, P = .001 and P = .001, respectively), ^g^CVMS-3 vs. CVMS-5 and CVMS-6 (P = .001, P = .001, respectively), ^h^CVMS-4 vs. CVMS-5 and CVMS-6 (P = .007, P = .001, respectively)

*Significant at P < .05

*CVMS: Cervical Vertebral Maturation Stages*

The study sample had a mean age of 12.68 ± 2.65 years. A significant age difference was observed between girls and boys (11.69 ± 2.54 years for girls versus 13.66 ± 2.40 years for boys).

There was no significant difference by sex among HWMS and CVMS.

The intraclass correlation coefficient is considered to be high in the range of 0.80–0.94, while it is considered excellent in the range 0.95- 1.00 [29]. The intra-observer agreement was examined, which ranged from high to excellent **(**Table 4**)**.

**Table 4:** Results of statistical analysis for intra-observer agreement by ROI

	Intra-class correlation coefficient	P value	
Proximal Phalanx	0.827	0.001*	
Medial Phalanx	0.898		
Distal Phalanx	0.899		
Radius	0.961		
C2	0.804		
C3	0.926		
C4	0.984		

*Significant at P < .05

## Discussion

Many studies have reported that since skeletal development stages can exhibit a wide range of chronological age, skeletal maturation stages assessed by radiographic measurements may provide more accurate data than chronological age [31, 32]. However, evaluation of skeletal maturation stages is entirely based on morphological examination, which may be associated with poor reproducibility and poses a challenge for orthodontists to conclusively determine the maturation stage of an individual [33]. In such cases, being a quantitative method, fractal analysis may provide more objective data.

In this study investigating the correlation of FD measurements from the regions of interest with skeletal maturation stages, significant weak or moderate positive correlations were found between all FD measurements except distal phalanx from hand-wrist radiographs and HWMS or CVMS. These results suggest that changes occur in the internal trabecular structure of the bones which were morphologically examined to determine skeletal maturation stage. In other words, as the skeletal maturation stage increases, complexity of the internal trabecular pattern seems to increase, resulting in a greater fractal dimension value.

In a study by Akbulut et al. [20] comparing FD values of the third finger phalanges and the radius according to the outcome of rapid palatal expansion (RPE), lower FD values were reported in the medial and distal phalanges and radius in patients with succesful RPE outcomes compared to those with failed RPE. Also, the FD value of the radius was positively correlated with age and HWMS in this study [20]. They suggested that FD values from hand-wrist radiographs may provide useful information for predicting the success of RPE surgery and that the epiphyseal-diaphyseal line of the radius can provide more robust data on overall skeletal maturation in postpubertal individuals.

In the current study which included a larger sample, FD values from the medial phalanx, proximal phalanx and radius were significantly different among HWMS and CVMS. The fractal results measured from these regions were found significantly higher in the higher stages compared to lower stages. The significant results obtained from these analyses support that FD values increase with skeletal maturation. This suggests that the fractal analysis method can provide guidance for clinical decisions in the case when skeletal maturation stage cannot be definitively determined.

Morphological alterations that occur in the epiphyseal and diaphyseal lines and narrowing of the joint space due to the convergence of epiphysis and diaphysis with increasing age may have led to increased FD values as found in the present study. When the sutures in the epiphyseal and diaphyseal lines fuse, the joint space disappears and the FD value is likely to increase due to increased suture complexity [34]. It has been reported that a narrower joint space is associated with more complex suture morphology [35]. The findings of the current study suggest that the radius FD values may be useful for determining the maturation stage. As reported in the literature, changes in ROI size may affect FD values [36]. Although the ROI size was not standardized in Akbulut et al.’s study [20], significant results were observed for the radius FD values, consistent with the current study findings.

In the present study, weak but positive correlations were shown between the C2 FD values and age or HWMS and between the C3 FD values and age, HWMS or CVMS (P < .05). In addition, a moderate correlation was found between C3 and C4 FD values. As opposed to the current study, no statistically significant correlations were found between FD values of the cervical vertebrae (C2, C3, C4) and chronological age, HWMS or CVMS in a study by Pamukçu et al. investigating the correlation of FD measurements from the cervical vertebrae with HWMS and CVMS on radiographs of 120 subjects [13]. However, again in contrast to the current study, when HWMS and CVMS were divided into two groups according to pubertal growth spurt (PGS) as pre-PGS and post-PGS, significant negative correlations were found between C4 FD values and HWMS or CVMS both in females and in the entire sample in the same study. Based on this finding, the authors suggested that the C4 FD value can be used as an objective tool to predict skeletal maturation stage and especially pubertal growth spurt. The limited sample size, the absence of testing for intra-observer agreement and uneven distributions of sex and patients among the stages may have accounted for the different results reported by Pamukçu et al. [13].

When the FD values measured from hand-wrist and lateral cephalometric radiographs were compared, it was found that the FD values of the radius were positively but weakly correlated with those of all vertebrae (C2, C3, C4) examined in this study (P < .05). This suggests that the radius develops parallel to the cervical vertebrae during the skeletal maturation process. In addition, the radius FD values were also weakly correlated with the FD values of the proximal and medial phalanges and age (P < .05). Positive moderate correlations were also observed between the radius FD values and HWMS or CVMS, with significant differences in the radius FD values among HWMS and CVMS (P < .05). These findings support Akbulut et al.’s argument [20] that the radius FD values may provide precise information about skeletal maturation stage. In this study, the significant results and correlations between the FD values measured especially from the radius, indicate the necessity to focus more on the radius in future research.

Similar to the current study which demonstrated very strong positive correlations between HWMS and CVMS, several studies reported a high-level agreement between HWM and CVM methods [13–15]. The results of a systematic review and meta-analysis of 19 studies showed positive correlations between HWM (irrespective of the method used) and CVM assessments (Hassel-Farman), which were maintained when the analysis was performed with (higher correlation for females versus males (r = .925 and r = .879, respectively) or without (r = .592) gender saparation [16]. However, Beit et al. [37] reported that the CVM method is not reliable in predicting skeletal age.

In the current study, age showed strong positive correlations with HWMS and CVMS in both girls (r = .905, r = .861, respectiviely) and boys (r = .824, r = .783, respectively) (P < .05). Consistently, Pamukcu et al. [13] found strong positive correlations of chronological age with both HWMS (r = .831 for girls, r = .932 for boys and overall r = .831) and CVMS (r = .795 for girls, r = .825 for boys and overall r = .793).

In the present study, while the sex distribution was homogenous among the groups, the mean age of the boys was significantly higher compared to girls (P < .001). This can be explained by the fact that compared to boys, skeletal maturation is much faster and pubertal growth spurt occurs 1 2 years earlier in girls [13].

The only limitation of this study was the inability to collect a sufficient number of radiographs representing HWMS 1 and 2 due to often not initiating orthodontic treatment in prepubertal children at HWMS 1 and 2. In addition, artifacts were frequently encountered in the radiographs of young children at these developmental stages. There is a need for further studies evaluating skeletal maturation, especially of the radius, in a larger sample size, including the underrepresented prepubertal age groups.

## Conclusions

Moderate and weak positive correlations and significant differences were found between FD measurements obtained from hand-wrist and lateral cephalometric radiographs and between these measurements and HWMS or CVMS. When compared with other regions, FD values measured from the radius appear to be more reliable to predict skeletal maturation in growing individuals, as shown by a greater number of correlations and significant results with FD values measured from other regions, CVMS, HWMS and age. Especially, the FD values from the radius can provide valuable guidance for the treatment of skeletal disorders where the determination of maturation is crucial.

## Data Availability

The datasets created and/or analyzed during the current study are not publicly available due to [Ethics committee decision], but are available from the corresponding author upon reasonable request.

## References

[1] Xavier AM, Chandra HS, Vijay M. Craniofacial Growth and Development in Children. In: Damle S, Kalaskar R, Sakhare D, Jetpurwala A, editors. Illustrated Pediatric Dentistry (Part I). Bentham Books; 2022. pp. 192–221.

[2] Byun B-R, Kim Y-I, Yamaguchi T, Maki K, Ko C-C, Hwang D-S (2015). Quantitative skeletal maturation estimation using cone-beam computed tomography-generated cervical vertebral images: a pilot study in 5-to 18-year-old japanese children. Clin Oral Investig.

[3] Thevissen P, Kaur J, Willems G (2012). Human age estimation combining third molar and skeletal development. Int J Legal Med.

[4] Eninanç İ, Yeler DY, Çınar Z (2021). Investigation of mandibular fractal dimension on digital panoramic radiographs in bruxist individuals. Oral surg. Oral Med. Oral Pathol Oral Radiol.

[5] Baccetti T, Franchi L, McNamara JA (2005). The cervical vertebral maturation (CVM) method for the assessment of optimal treatment timing in dentofacial orthopedics. Semin Orthod.

[6] Flores C, Nebbe B, Major PW (2004). Use of skeletal maturation based on hand-wrist radiographic analysis as a predictor of facial growth: systematic review. Angle Orthod.

[7] Buschang P, Roldan S, Tadlock L (2017). Guidelines for assessing the growth and development of orthodontic patients. Semin Orthod.

[8] Patil N, Maheshwari N, Sharma R, Soni S, Kushwah A (2019). Correlation between chronological age, cervical vertebral maturation and Fishman’s skeletal maturity indicators in Central India Population. Orthod J Nepal.

[9] Bunch PM, Altes TA, McIlhenny J, Patrie J, Gaskin CM (2017). Skeletal development of the hand and wrist: digital bone age companion—a suitable alternative to the Greulich and Pyle atlas for bone age assessment?. Skeletal Radiol.

[10] Srinivasan B, Padmanabhan S, Chitharanjan AB (2018). Constancy of cervical vertebral maturation indicator in adults: a cross-sectional study. Int Orthod.

[11] Hassel B, Farman AG (1995). Skeletal maturation evaluation using cervical vertebrae. Am J Orthod Dentofacial Orthop.

[12] Baccetti T, Franchi L, McNamara JA (2002). An improved version of the cervical vertebral maturation (CVM) method for the assessment of mandibular growth. Angle Orthod.

[13] Pamukcu U, Ispir NG, Akay G, Karadag Atas O, Gungor K, Toraman M (2022). Evaluation of the compatibility of C2, C3, and C4 fractal dimension values with hand-wrist and cervical vertebra maturation methods in determining skeletal maturation. Dentomaxillofac Radiol.

[14] Tekin A, Cesur Aydın K (2020). Comparative determination of skeletal maturity by hand–wrist radiograph, cephalometric radiograph and cone beam computed tomography. Oral Radiol.

[15] Jeon JY, Kim C-S, Kim J-S, Choi S-H (2021). Correlation and correspondence between skeletal maturation indicators in hand-wrist and cervical vertebra analyses and skeletal maturity score in korean adolescents. Children.

[16] Cericato G, Bittencourt M, Paranhos L (2015). Validity of the assessment method of skeletal maturation by cervical vertebrae: a systematic review and meta-analysis. Dentomaxillofac Radiol.

[17] Durka-Zając M, Marcinkowska A, Mituś-Kenig M (2013). Bone age assessment using cephalometric photographs. Pol J Radiol.

[18] Joshi V, Yamaguchi T, Matsuda Y, Kaneko N, Maki K, Okano T (2012). Skeletal maturity assessment with the use of cone-beam computerized tomography. Oral Surg Oral Med Oral Pathol Oral Radiol.

[19] Sánchez I, Uzcátegui G (2011). Fractals in dentistry. J Dent.

[20] Akbulut S, Bayrak S, Korkmaz YN (2020). Prediction of rapid palatal expansion success via fractal analysis in hand-wrist radiographs. Am J Orthod Dentofacial Orthop.

[21] Wolski M, Podsiadlo P, Stachowiak GW (2014). Directional fractal signature methods for trabecular bone texture in hand radiographs: data from the Osteoarthritis Initiative. Med Phys.

[22] Jurczyszyn K, Kubasiewicz-Ross P, Nawrot-Hadzik I, Gedrange T, Dominiak M, Hadzik J (2018). Fractal dimension analysis a supplementary mathematical method for bone defect regeneration measurement. Ann Anat.

[23] Zandieh S, Haller J, Bernt R, Hergan K, Rath E (2017). Fractal analysis of subchondral bone changes of the hand in rheumatoid arthritis. Med.

[24] Sanchez-Molina D, Velazquez-Ameijide J, Quintana V, Arregui-Dalmases C, Crandall JR, Subit D (2013). Fractal dimension and mechanical properties of human cortical bone. Med Eng Phys.

[25] Satoh M (2015). Bone age: assessment methods and clinical applications. Clin Pediatr Endocrinol.

[26] Björk A. Timing of interceptive orthodontic measures based on stages of maturation. Trans Europ Orthod Soc 1972:61–74. 4363429

[27] Grave K, Brown T (1976). Skeletal ossification and the adolescent growth spurt. Am J Orthod.

[28] White SC, Rudolph DJ (1999). Alterations of the trabecular pattern of the jaws in patients with osteoporosis. Oral Surg Oral Med Oral Pathol Oral Radiol Endod.

[29] Alpar R, Spor S (2016). Applied Statistics and validity-reliability with examples from Educational Sciences.

[30] Sümbüloğlu K, Sümbüloğlu V (2012). Biostatistics.

[31] Baccetti T, Franchi L, McNamara JA (2005). The cervical vertebral maturation (CVM) method for the assessment of optimal treatment timing in dentofacial orthopedics. Semin Orthod.

[32] Fishman LS (1982). Radiographic evaluation of skeletal maturation: a clinically oriented method based on hand-wrist films. Angle Orthod.

[33] Nestman TS, Marshall SD, Qian F, Holton N, Franciscus RG, Southard TE (2011). Cervical vertebrae maturation method morphologic criteria: poor reproducibility. Am J Orthod Dentofacial Orthop.

[34] Byron CD (2006). Role of the osteoclast in cranial suture waveform patterning. Anat Rec a Discov Mol Cell Evol Biol.

[35] Hartwig WC (1991). Fractal analysis of sagittal suture morphology. J Morphol.

[36] Shrout M, Farley B, Patt S, Potter B, Hildebolt C, Pilgram T (1999). The effect of region of interest variations on morphologic operations data and gray-level values extracted from digitized dental radiographs. Oral Surg Oral Med Oral Pathol Oral Radiol Endod.

[37] Beit P, Peltomäki T, Schätzle M, Signorelli L, Patcas R (2013). Evaluating the agreement of skeletal age assessment based on hand-wrist and cervical vertebrae radiography. Am J Orthod Dentofacial Orthop.

